# Auxin-induced Rapid Degradation of Inhibitor of Caspase-activated DNase (ICAD) Induces Apoptotic DNA Fragmentation, Caspase Activation, and Cell Death: A CELL SUICIDE MODULE*

**DOI:** 10.1074/jbc.M114.583542

**Published:** 2014-09-23

**Authors:** Kumiko Samejima, Hiromi Ogawa, Alexander V. Ageichik, Kevin L. Peterson, Scott H. Kaufmann, Masato T. Kanemaki, William C. Earnshaw

**Affiliations:** From the ‡ Wellcome Trust Centre for Cell Biology, University of Edinburgh, King's Buildings, Max Born Crescent, Edinburgh EH9 3BF, Scotland, United Kingdom,; the § Mayo Clinic, Rochester, Minnesota 55905,; the ¶ Centre for Frontier Research, National Institute of Genetics, ROIS, and Department of Genetics, SOKENDAI, Yata 1111, Mishima, Shizuoka 411-8540, Japan, and; the ‖ Japan Science and Technology Agency (JST), PREST, 4-1-8 Honcho, Kawaguchi, Saitama 332-0012, Japan

**Keywords:** Apoptosis, Auxin, Caspase, Cell Death, Yeast, Annexin V, Caspase-activated DNase (CAD), DNA Fragmentation, Inhibitor of Caspase-activated DNase (ICAD), TUNEL Assay

## Abstract

Caspase-activated DNase (CAD) is a major apoptotic nuclease, responsible for DNA fragmentation and chromatin condensation during apoptosis. CAD is normally activated in apoptosis as a result of caspase cleavage of its inhibitory chaperone ICAD. Other aspects of CAD regulation are poorly understood. In particular, it has been unclear whether direct CAD activation in non-apoptotic living cells can trigger cell death. Taking advantage of the auxin-inducible degron (AID) system, we have developed a suicide system with which ICAD is rapidly degraded in living cells in response to the plant hormone auxin. Our studies demonstrate that rapid ICAD depletion is sufficient to activate CAD and induce cell death in DT40 and yeast cells. In the vertebrate cells, ectopic CAD activation triggered caspase activation and subsequent hallmarks of caspase-dependent apoptotic changes, including phosphatidylserine exposure and nuclear fragmentation. These observations not only suggest that CAD activation drives apoptosis through a positive feedback loop, but also identify a unique suicide system that can be used for controlling gene-modified organisms.

## Teaser

**Background:** Regulation and function of the apoptotic nuclease, caspase-activated DNase (CAD), remains obscure.

**Results:** Rapid depletion of the CAD inhibitor (ICAD) using auxin-inducible degron system induces ectopic CAD activation and apoptosis.

**Conclusion:** Ectopic CAD activation triggers caspase activation and apoptosis through a positive feedback loop.

**Significance:** Controlled CAD dissociation from ICAD is applicable as a novel method to eliminate unwanted cells.

## Introduction

Apoptosis is a cell-autonomous pathway that eliminates unwanted or damaged cells without harming neighboring tissues. Apoptotic proteases, most notably caspases, play important roles in the pathways leading to apoptotic execution (1–4). To prevent accidental activation of caspases and cell death, cells have evolved elaborate regulatory mechanisms to regulate caspase activity (1, 2, 4–9). In addition to apoptotic proteases, apoptotic nucleases typically function during the execution phase of apoptosis (10, 11). Importantly, however, the regulation of apoptotic nucleases is less well understood. Although the action of the caspase-activated DNase (CAD)^5^ nuclease is sufficient to drive nuclear events of classical apoptosis such as DNA fragmentation and apoptotic chromatin condensation (12), the possibility that activity of this endogenous nuclease by itself is capable of initiating the death of healthy living cells has never been tested.

**FIGURE 1.**
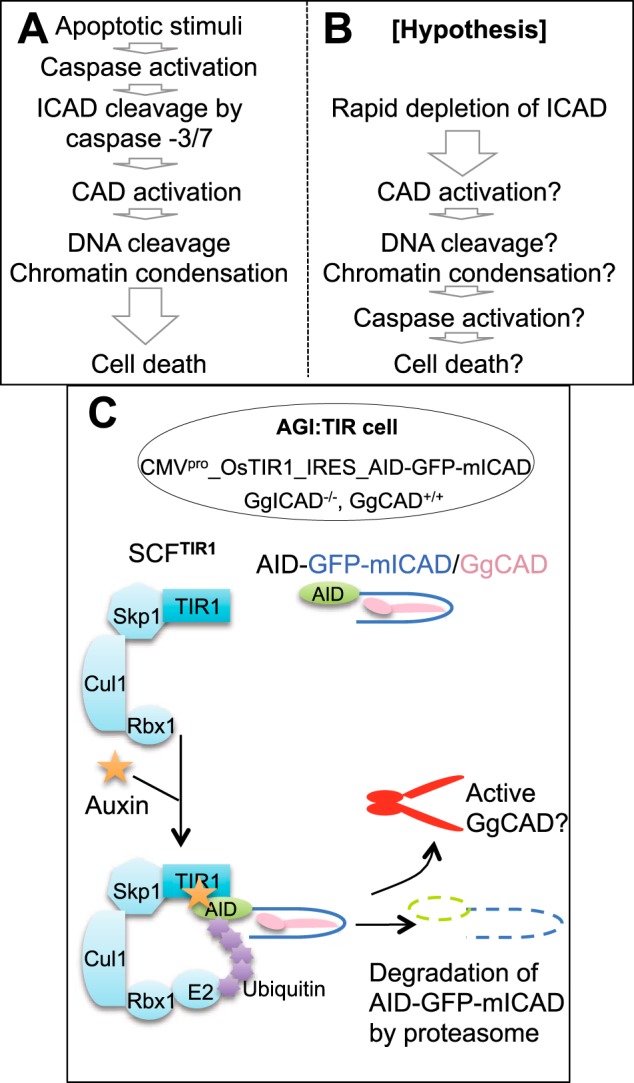
**Schematic of our experimental system.** *A*, conventional apoptotic pathway. *B*, hypothesis: Does rapid depletion of ICAD-L trigger CAD activation and cell death? *C*, experimental system using AGI:TIR1 cells. A plasmid encoding OsTIR1 and AID-GFP-mICAD-L was transfected into ICAD^−/−^CAD^+/+^ chicken DT40 cells. F-box protein OsTIR1 binds to endogenous Skp1 to form the SCF^TIR1^(Skp1-Cullin1-TIR1) complex. TIR1 binds to an AID tag in the presence of auxin (27). SCF^TIR1^ ubiquitinates the AID tag and promotes the degradation of AID-tagged ICAD protein by proteasome. We set out to test whether rapid degradation of ICAD could activate CAD.

In this study, we describe a synthetic suicide module that is based on an apoptotic nuclease and can be induced by the addition of the ubiquitous plant hormone indoleacetic acid (auxin). This cell suicide module utilizes the major apoptotic nuclease, CAD (also known as DFF40) (13, 14). The inhibitor of CAD (ICAD/DFF45) normally forms an inactive complex with CAD. ICAD has dual roles as a chaperone required for CAD activity and as an inhibitor that prevents spontaneous CAD activation in living cells (14). During apoptotic execution (see Fig. 1*A*), caspase-3 normally cleaves ICAD at two sites (15). This liberates CAD, allowing it to form an active homo-dimer (16). Active dimeric CAD cleaves between nucleosomes, inducing DNA fragmentation and the final stages of apoptotic chromatin condensation (12). Cells are viable without CAD, and it has been reported that loss of CAD activity can increase cell survival. Conversely, CAD overexpression can increase cell death (17–19). Treatment of acute myeloid leukemia cells with purified CAD fused with granulocyte/macrophage colony-stimulating factor induced apoptosis (20). Moreover, CAD activation has been linked to cell death during prolonged mitotic arrest (21). These studies suggest that ectopic CAD activation in living cells might induce cell death. However, whether there is a safety mechanism apart from ICAD to prevent ectopic CAD activation in living cells has been unclear.

We previously modified ICAD to replace caspase cleavage sites with tobacco etch virus protease cleavage sites (22). Treatment of the modified ICAD with tobacco etch virus protease led to CAD activation *in vitro*, in *Escherichia coli*, and in yeast (where endogenous CAD/ICAD are absent) (22, 23). Knowing that CAD can be activated by ICAD degradation independent of caspases, we decided to test whether proteasomal degradation of ICAD *in situ* is sufficient to activate CAD and to induce cell death in healthy non-apoptotic cells (see Fig. 1, *B* and *C*).

## EXPERIMENTAL PROCEDURES

### 

#### 

##### Cell Culture and Transfections

The chicken lymphoma B cell line DT40 and its ICAD^−/−^ knock-out derivative (in which the entire ICAD ORF is deleted) were cultured as described previously (22, 24). Transfection to obtain AID-GFP-mICAD-expressing ICAD knock-out cells (AGI:TIR) was performed as described previously and selected by the addition of 1.5 mg/ml G418 (25).

##### AID Antibody Generation

cDNA encoding amino acids 28–102 of the *Arabidopsis thaliana* IAA17 protein fused to a His_6_ tag in the pET28c vector was transformed into *E. coli* BL21 codon plus. After isopropyl-β-d-1-thiogalactopyranoside induction, the protein was isolated on Ni^2+^-agarose, dialyzed at 4 °C into calcium- and magnesium-free Dulbecco's PBS, cross-linked by the addition of formaldehyde to 1% for 1 h at 4 °C, and dialyzed further in PBS to remove unreacted formaldehyde. Using this cross-linked antigen, murine hybridomas that secrete anti-AID antibody were generated as described in previous studies (26) using the Mayo Clinic Hybridoma Core Facility. Primary screening of culture supernatants was performed by ELISA using non-cross-linked His_6_-IAA17 (amino acids 28–102), and secondary screening was performed by immunoblotting as described below.

##### Subcloning, Antibodies, and Drug Treatments

GFP-mICAD-L (12) was cloned into pMK102 (27) using EcoRV and EcoRI sites. Antibodies used for immunoblotting and indirect immunofluorescence analysis were our mouse monoclonal anti-AID tag at 1:1000, rabbit anti-GFP at 1:1000 (Molecular Probes, Life Technologies), and mouse anti-tubulin B512 (Sigma) at 1:4000. Drugs (final concentration) used were auxin (indoleacetic acid) at 125 μm (Q-Val-Asp-CH_2_-OPh, non-*O*-methylated) in ethanol, etoposide at 10 or 100 μm (Calbiochem) in DMSO, and the caspase inhibitor at 10 or 50 μm (Calbiochem) in DMSO.

##### Indirect Immunofluorescence of AGI:TIR Cells

Cells were attached to polylysine-coated slides, fixed in 4% paraformaldehyde/PBS, permeabilized with 0.15% Triton X-100 for 2 min, and blocked with 1% BSA/PBS. Anti-AID antibody (1:1000 in blocking buffer) and anti-mouse secondary antibody (Alexa Fluor 594, 1:1000 from Molecular Probes) were used to visualize the tagged protein. Three-dimensional data sets were acquired using a cooled CCD camera (CoolSNAP HQ; Photometrics) on a wide-field microscope (DeltaVision RT; Applied Precision) with a 100× NA 1.4 Plan Apochromat lens. The data sets were deconvolved with softWoRx (Applied Precision), exported as TIFF files, and imported into Adobe Photoshop for final presentation.

##### Flow Cytometry

GFP-positive and -negative living cells were sorted using a FACSAria (BD Biosciences), and analysis was performed using FACSCalibur or LSRII (BD Biosciences) flow cytometers.

##### Annexin V Assay

Cell death was quantified using an annexin V-phycoerythrin (PE)-CY5 apoptosis detection kit (BioVision) following the manufacturer's instructions.

##### TUNEL Assay

DNA fragmentation was assessed using an *In Situ* cell death detection kit, TMR red (Roche Diagnostics GmbH, Mannheim Germany) for analysis with microscope or Click-iT TUNEL Alexa Fluor 647 (Life Technologies) for flow cytometry analysis following the manufacturer's instructions. For time course analysis, ∼1 × 10^6^ cells/sample were collected and fixed with 4% formaldehyde and then permeabilized with 0.25% Triton X-100.

##### Genomic DNA-Agarose Gel Electrophoresis

1 × 10^7^ cells/sample were treated with indoleacetic acid or 10 μm etoposide for 6 h. Cells were lysed in lysis buffer (200 mm Tris-HCl pH 7.4, 200 mm EDTA, 1% Nonidet P-40) for 10 s and centrifuged for 5 min to obtain the supernatant. After SDS was added (final: 1% SDS), samples were treated with proteinase K (final 2.5 μg/ml) overnight at 37 °C. Genomic DNA was precipitated with 1/10 volumes of 10 m ammonium acetate and 2.5 volumes of ethanol. The precipitate was washed with 70% ethanol, and the final precipitate was dissolved in Tris-EDTA (TE) buffer containing 5 μg/ml RNase overnight at 4 °C. Genomic DNA was loaded on 2% Tris-acetate-EDTA (TAE) agarose gels. DNA was stained with ethidium bromide.

##### Colony Formation Assay for DT40 Cells

Cells were treated for 6 h in the absence or presence of auxin, diluted, and plated in 96-well dishes so that each well contained one living cell. After 1–2 weeks, colonies (positive wells) were counted.

##### Caspase Activation Assay

3 × 10^5^cells/sample were treated with indoleacetic acid for 0–6 h in the presence of absence of 10 μm caspase inhibitor Q-VD-OPh. Caspase activation was analyzed using the FLICA 660 *in vitro* poly caspase detection kit (ImmunoChemistry Technologies LLC) following the manufacturer's instructions. In our case, cells were incubated with FLICA 660 dye for 1 h.

##### Yeast Strain Expressing AID-ICAD/CAD

*Saccharomyces cerevisiae* (strain BY25602: Genotype MATa ura3-1::GAL-OSTIR1-9myc(URA3)ade2-1 his3-11,15 lue2-3,112trp1-1 can1-100) was obtained from the Yeast Genetic Resource Centre, Osaka, Japan. HA-tagged mCAD (12) was amplified by PCR using primers (CTGAATTCGATGTGCGCGGTGCTC and CTGATATCTCACTAGCGCTTCCG), cloned into the EcoRI and EcoRV sites of the pYM-N36 plasmid (MET25 promoter: HA-mCAD), again amplified by PCR with primers (ACATGTATATATATCGTATGCTGCAGCTTTAAATAATCGGGTGTCATCACTAGCGCTTCCGAGCAG and AAGAATATACTAAAAAATGAGCAGGCAAGATAAACGAAGGCAAAGGACATGGAGGCCCAGAATACC), and then integrated into the His3 locus. HA-tagged mICAD-L (12) was amplified by PCR using primers (GGGCCCGGAGCTGGTGCAGGCGCTGGCCGCATCTTTTAC and GGTACCCTACGAGGAGTCTCG), cloned into the ApaI and KpnI sites of the pNHK12 plasmid (alcohol dehydrogenase I (ADH) promoter: AID-HA-mICAD-I), linearized by MfeI, and then integrated into Trp1 locus.

##### Colony Formation Assay for Yeast

The engineered *S. cerevisiae* cells were grown overnight in YPR, then diluted in YPR/YPG medium to *A*_600_ = 0.5. YPG medium induces OsTIR1 expression. Cells were grown for 1 h at 30 °C and diluted in water to *A*_600_ = 0.3, 0.03, or 0.003. Cells were replicate-plated on the following plates: YPR ± auxin and YPG ± auxin and incubated at 30 °C for 2 days to allow colony formation.

## RESULTS

**FIGURE 2.**
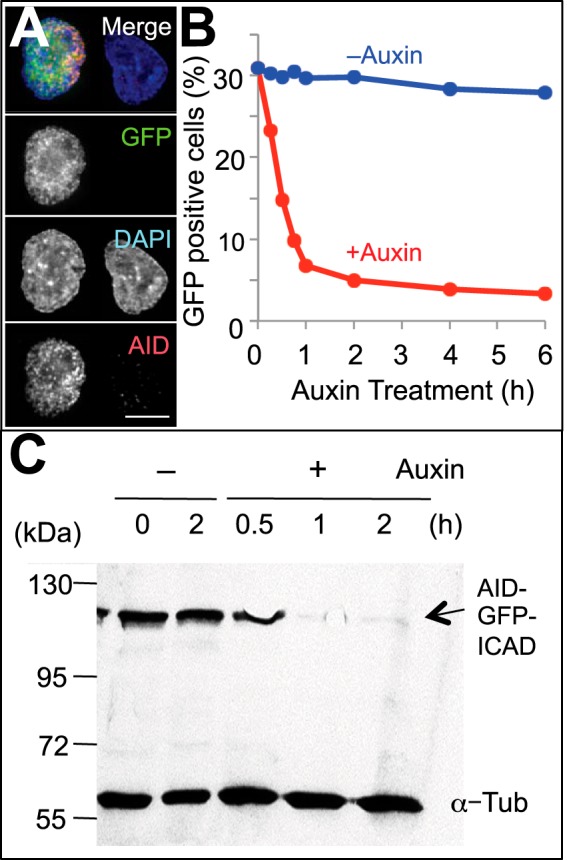
**Addition of auxin induces rapid depletion of AID-mICAD-L *in vivo*.** *A*, paraformaldehyde-fixed AID-GFP-mICAD-L-expressing cells (AGI:TIR) were stained with anti-AID mouse monoclonal antibody. *B*, the fraction of GFP-positive cells was detected by flow cytometry after the addition of 125 μm auxin. *C*, depletion of AID-GFP-mICAD-L protein after the addition of auxin was assessed by anti-GFP antibody. α-Tubulin (α-*Tub*) was a loading control.

To test our hypothesis that rapid destruction of ICAD is sufficient to activate CAD and lead to cell death, we utilized an auxin-inducible degron (AID) system (Fig. 1*C*) (27). We replaced the endogenous chicken (*Gallus gallus*) ICAD-L/S with AID-GFP-mICAD-L, a cassette that contains the AID degron fused to GFP (to monitor expression) and murine ICAD-L, taking advantage of ICAD^−/−^ knock-out chicken DT40 cells that we established previously (22). In those cells, the AID-GFP-mICAD-L is the only source of ICAD and regulates the activity of endogenous chicken CAD. OsTIR1, a plant-specific F-box protein, was further incorporated into those cells, which we termed AGI:TIR (**A**ID-**G**FP-m**I**CAD-L:**TIR**1) cells. OsTIR1 binds to endogenous Skp1, forming the SCF^TIR1^ ubiquitin ligase complex. The expression and correct localization of tagged ICAD-L were confirmed using anti-AID monoclonal antibody and GFP fluorescence (Fig. 2, *A* and *C*).

**FIGURE 3.**
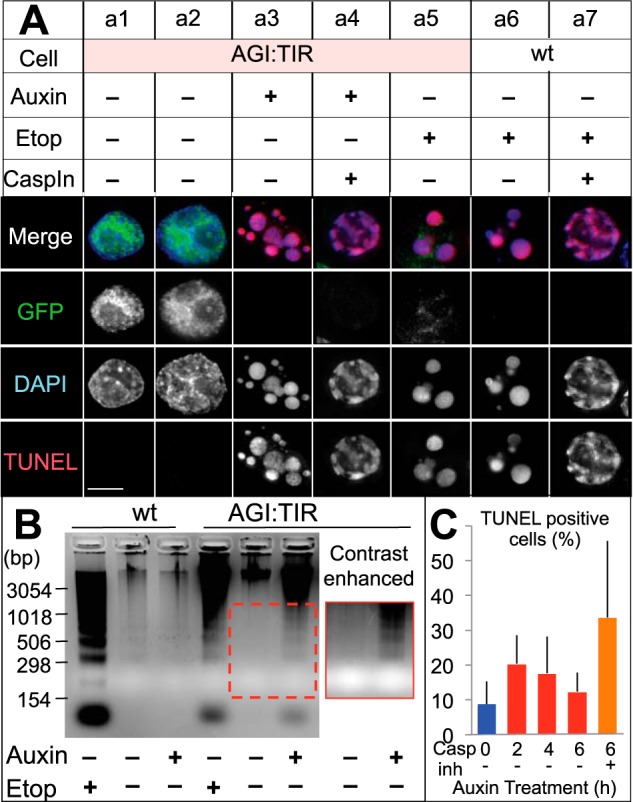
**Rapid degradation of ICAD-L is capable of activating CAD *in vivo*, leading to DNA fragmentation and apoptotic chromatin condensation.** *A*, TUNEL staining of the AGI:TIR cells at time = 0 (*column a1*) or after a 5-h treatment with solvent (*column a2*), auxin (*column a3*), auxin + caspase inhibitor (*column a4*), or etoposide (*Etop*; *column a5*); wild type cells treated with etoposide (*column a6*); or wild type cells treated with etoposide and caspase inhibitor (*column a7*). *B*, wild type DT40 cells or the AGI:TIR cells were treated with etoposide or auxin for 5 h. Internucleosomal DNA was visualized by agarose gel electrophoresis. A contrast-enhanced picture is shown within the *red boxed region. C*, flow cytometry analysis of TUNEL assay at various time points after the addition of auxin to the AGI:TIR1 cells. *Bars* show S.D. (*n* = 3). *Casp inh*, caspase inhibitor.

The addition of auxin to AGI:TIR cells induced rapid depletion of AID-GFP-tagged ICAD, leading to CAD activation, caspase activation, and apoptosis. The number of GFP-positive AGI:TIR cells declined within 1 h after auxin addition (Fig. 2*B*). In parallel, speedy depletion of AID-GFP-mICAD-L protein was observed by immunoblotting (Fig. 2*C*). This resulted in CAD activation as demonstrated by the appearance of TUNEL-positive cells and the formation of characteristic apoptotic bodies (Fig. 3*A*, *column a3*, and 3*C*). Apoptotic chromatin condensation in auxin-treated AGI:TIR cells was morphologically indistinguishable from that in their counterparts treated with etoposide, a drug known to induce apoptosis (Fig. 3a5) (28, 29), as well as wild type cells treated with etoposide (Fig. 3*A*, *column a6*). Comparable levels of internucleosomal DNA cleavage were observed in AGI:TIR cells treated with either auxin or etoposide (Fig. 3*B*). In contrast, no DNA fragmentation was observed in auxin-treated wild type cells. Somewhat more pronounced DNA fragmentation was detected in etoposide-treated wild type cells as compared with AGI:TIR cells as discussed below.

To determine the time course of CAD activation following loss of ICAD, we employed a TUNEL assay to directly measure CAD function. The ratio of TUNEL-positive cells increased from 8.4 to 20.5% during the initial 2 h after auxin addition, indicating that CAD activation occurs within the first 2 h. The ratio of TUNEL-positive cells subsequently decreased to 17.7% (4 h) and 12.4% (6 h). This was unexpected and is likely explained by the loss of cleaved DNA from the apoptotic cells during centrifugation steps. If apoptosis was blocked by the addition of a caspase inhibitor, 33.7% of cells remained TUNEL-positive after a 6-h auxin treatment. This result demonstrates that CAD activation occurred following proteasome-mediated destruction of ICAD in AGI:TIR cells.

**FIGURE 4.**
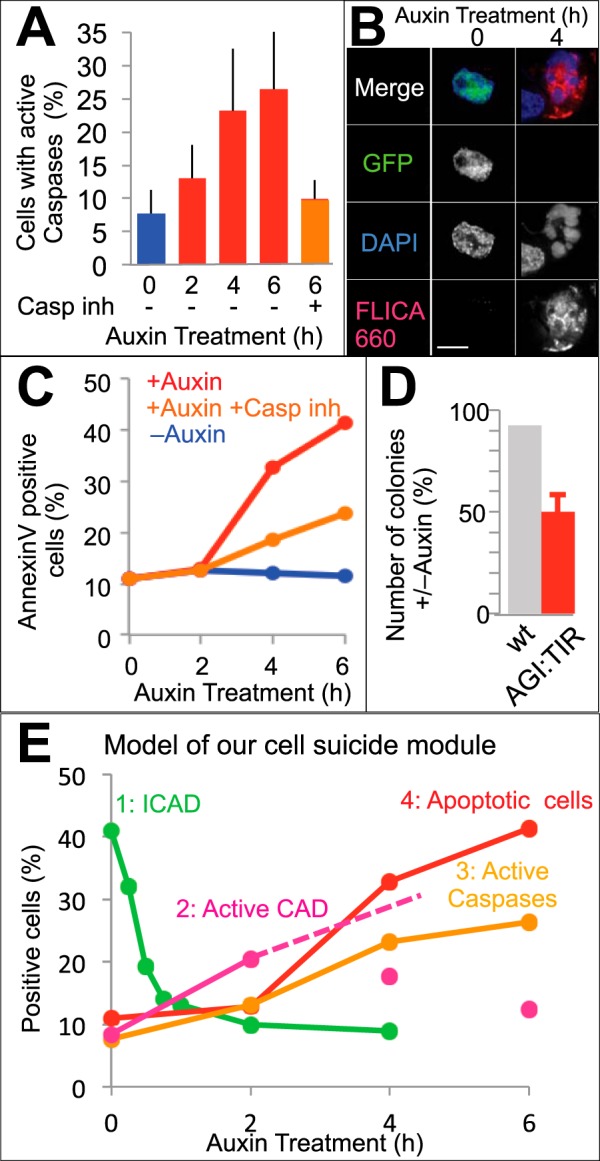
**Ectopic activation of CAD is sufficient to induce caspase activation and cell death.** *A* and *B*, caspase activation detected by FLICA 660 *in vitro* caspase detection kit. AGI:TIR cells were treated with the indicated drugs and analyzed by flow cytometry (*A*) or by microscopy (*B*). *Bars* show S.D. (*n* = 3). *Casp inh*, caspase inhibitor. *C*, cell death detected by annexin V assay. The AGI:TIR cells were treated with the indicated drugs and analyzed by flow cytometry. *D*, long term cell survival was examined in a colony formation assay. Ratios between cells (either wild type or AGI:TIR cells) treated with auxin or not are shown. *Bars* show S.D. (*n* = 3). *E*, time course of events following activation of the cell suicide module.

CAD normally functions downstream of caspases during apoptotic execution (Fig. 1*A*). We therefore asked whether CAD activation can subsequently lead to caspase activation, thereby triggering the full activation of the apoptotic machinery (Fig. 1*B*). Indeed, caspases became progressively activated in AGI:TIR cells from 2 h after auxin addition (Fig. 4, *A* and *B*). This caspase activation was almost entirely suppressed by the addition of 10 μm caspase inhibitor (Fig. 4*A*).

We further examined CAD-induced changes using additional assays for cell death. Annexin V has a high affinity for phosphatidylserine, which appears on the outer leaflet of the plasma membrane in a caspase-dependent manner during apoptosis in many cell types (30–33). Indeed, an increase in annexin V binding was detectable within 4 h after the addition of auxin to AGI:TIR cells, and nearly half of the auxin-treated AGI:TIR1 cells became annexin V-positive by 6 h after the addition of auxin (Fig. 4*C*).

With the addition of the caspase inhibitor, the death of auxin-treated AGI:TIR cells was substantially suppressed (Fig. 4*C*), and apoptotic chromatin condensation remained at the rim stage while apoptotic body formation was suppressed (Fig. 3*A*, *column a4*). This mimicked the phenotype seen when etoposide and caspase inhibitor were added together to normal cells (Fig. 3A, *column a4 versus column 3a7*). These results strongly suggest that CAD activation triggers caspase activation in a positive feedback loop to promote apoptosis (Fig. 4*E*).

Clonogenic assays indicated that nearly half of the auxin-treated AGI:TIR cells failed to form colonies after auxin treatment, whereas fewer than 10% of the auxin-treated wild type cells were affected (Fig. 4*D*). It is important to note that the 50% decrease in colony formation in auxin-treated AGI:TIR cells is an underestimation of the toxicity of this module as discussed below.

**FIGURE 5.**
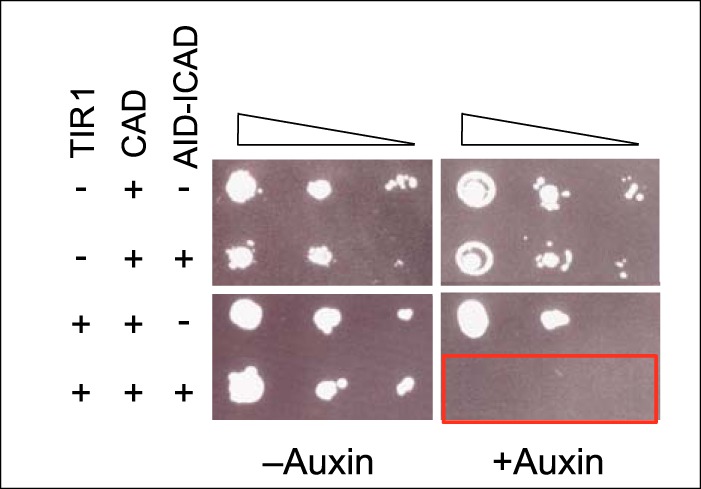
**Ectopic activation of CAD kills *S. cerevisiae*.** Serial dilutions of *S. cerevisiae* expressing the indicated elements were plated in the presence or absence of 125 μm indoleacetic acid.

After confirming our hypothesis using the chicken DT40 cell system, we decided to test whether a complete “AID-mICAD/mCAD plus OsTIR1” cell suicide module will work in a different organism, *S. cerevisiae* (Fig. 5). The auxin-inducible degron system was originally developed for use in *S. cerevisiae* (27). In addition, there is no homologue of CAD/ICAD in *S. cerevisiae*, which makes this organism ideal to test this cell suicide module.

Engineered yeast cells expressing AID-mICAD/mCAD plus OsTIR1 over a 100-fold range of concentrations were plated in the presence or absence of auxin. No colony appeared when the engineered yeast cells were plated in the presence of auxin. This demonstrates the effectiveness of the module even in a species that does not normally express CAD and ICAD.

## DISCUSSION

In the present work, we demonstrate for the first time that ectopic activation of the nuclease CAD is sufficient to induce caspase-dependent apoptosis. These observations not only place CAD both downstream and upstream of caspases during apoptotic execution, but also provide a novel strategy to assure eradication of genetically engineered organisms that are released into the environment.

Previous results placed the nuclease CAD downstream of executioner caspases during apoptotic cell demolition (Fig. 1*A*) (12, 14, 15). Our observations, however, show that destruction of ICAD by the proteasome in the continued presence of CAD leads to caspase activation (Fig. 4, *A* and *B*) and triggers hallmark changes of apoptosis, including phosphatidylserine exposure (Fig. 4*C*), chromatin condensation, and nuclear fragmentation (Fig. 3*A*, *column a3*). Both phosphatidylserine exposure and nuclear fragmentation are dependent on caspase-mediated cleavages (32–34), supporting that CAD activation can lead to downstream caspase activation. Consistent with this conclusion, we observed increased affinity labeling of active caspases after ICAD destruction (Fig. 4*A*). Importantly, the addition of a broad spectrum caspase inhibitor almost entirely suppressed the caspase activation (Fig. 4*A*) and decreased auxin-induced annexin V binding (Fig. 4*C*) and nuclear fragmentation (Fig. 3*A*, *column a4*).

Following ICAD destruction, we observed an initial rise in TUNEL-positive cells followed by a subsequent decline at the 4- and 6-h time points. We believe that this is a technical artifact of the TUNEL assay, which requires fixation and permeabilization of cells followed by repeated washing by centrifugation. Initial experiments describing DNA cleavage in apoptosis actually exploited this release of cleaved DNA from permeabilized apoptotic cells (35, 36), and we suggest that loss of the fragmented DNA from apoptotic cells could produce a decline in the TUNEL signal detected by flow cytometry. Consistent with this hypothesis, if we blocked the induction of apoptosis with a caspase inhibitor, the percentage of TUNEL-positive cells after ICAD destruction stayed high.

When all of our data are combined, the time course of induction of the cell suicide machinery can be recognized (Fig. 4*E*). After auxin addition, most ICAD was degraded by the proteasome within 1 h. This triggered CAD activation within 2 h. Subsequently, caspase activation was evident after 4 h, and this corresponded with an increase in cell death. These observations provide the first evidence for a positive feedback loop from CAD to caspases during apoptosis.

Additional experiments indicated that the “AID-GFP-mICAD plus OsTIR” module can eradiate unwanted cells. Our studies in yeast (Fig. 5) indicate that this system can kill cells even if they do not have an endogenous CAD/ICAD system. Parallel observations in vertebrate cells demonstrate that this system rapidly diminishes viability of AID-GFP-mICAD-expressing cells (Fig. 4). Our observation that 50% of cells lost the ability to form colonies within 6 h likely represents an underestimation of the efficacy of this module, as 70% of the cells expressed vanishingly small amounts of AID-GFP-mICAD by flow cytometry at the time of auxin addition (Fig. 2*B*). The residual cell survival and decreased extent of DNA fragmentation observed in AGI:TIR cells (Fig. 2*B*) likely arise from a reduced amount of ICAD expression in those cells. Indeed, we obtained no stable lines in which all cells were GFP-positive by flow cytometry analysis, and the GFP-positive population gradually declined in culture. We suspect that low level spontaneous cleavage of ICAD may provide a selection for cells that have silenced the module, a technical point that needs to be addressed in future experiments.

In summary, we have developed a cell suicide module consisting of AID-mICAD/mCAD plus OsTIR1, which works in both vertebrate cells and yeast. Our data demonstrate that rapid degradation of ICAD in vertebrate cells is sufficient to activate CAD, leading to apoptotic death. This killing is largely caspase-dependent, which indicates that there must be a positive feedback loop between activated CAD, DNA damage, and caspase activation. Importantly, our result is the first to show that ectopic activation of endogenous CAD can kill healthy, non-apoptotic cells. This suggests the possibility that the CAD/ICAD interaction could potentially be harnessed for elimination of unwanted cells. The ability to create synthetic organisms for use in biotechnology and genetically modified cells for use in potential therapies raises the important question of how those cells can be controlled once released among other normal cells or into the environment (37). We speculate that further developments outfitting genetically engineered organisms with this suicide module might provide a novel safety mechanism to eliminate such organisms if they are released into the normal environment where auxin is routinely present.

## Footnotes

5 The abbreviations used are:

CAD: caspase-activated DNase
ICAD: inhibitor of CAD
AID: auxin-inducible degron
DMSO: dimethyl sulfoxide
m: mouse.
